# qPCR-High resolution melt analysis for drug susceptibility testing of *Mycobacterium leprae* directly from clinical specimens of leprosy patients

**DOI:** 10.1371/journal.pntd.0005506

**Published:** 2017-06-01

**Authors:** Sergio Araujo, Luiz Ricardo Goulart, Richard W. Truman, Isabela Maria B. Goulart, Varalakshmi Vissa, Wei Li, Masanori Matsuoka, Philip Suffys, Amanda B. Fontes, Patricia S. Rosa, David M. Scollard, Diana L. Williams

**Affiliations:** 1 National Reference Center for Sanitary Dermatology and Leprosy, Federal University of Uberlandia, Uberlandia, Minas Gerais, Brazil; 2 Post-Graduate Program in Health Sciences, School of Medicine, Federal University of Uberlandia, Uberlandia, Minas Gerais, Brazil; 3 Division National Hansen's Disease Programs (NHDP), Healthcare Systems Bureau (HSB), Health Resources and Services Administration (HRSA), U.S. Department of Health and Human Services (DHHS), Baton Rouge, Louisiana, United States of America; 4 Institute of Genetics and Biochemistry, Federal University of Uberlandia, Uberlandia, Minas Gerais, Brazil; 5 Good Samaritan Society, Fort Collins, Colorado, United States of America; 6 Department of Microbiology, Immunology and Pathology, Colorado State University, Fort Collins, Colorado, United States of America; 7 Leprosy Research Center, National Institute of Infectious Diseases, Tokyo, Japan; 8 Fundação Oswaldo Cruz, Laboratory of Molecular Biology applied to Mycobacteria, Rio de Janeiro, Rio de Janeiro, Brazil; 9 Mycobacteriology Unit, Institute of Tropical Medicine, Antwerp, Belgium; 10 Instituto Lauro de Souza Lima, Department of Biology, Bauru, São Paulo, Brazil; University of Washington, UNITED STATES

## Abstract

**Background:**

Real-Time PCR-High Resolution Melting (qPCR-HRM) analysis has been recently described for rapid drug susceptibility testing (DST) of *Mycobacterium leprae*. The purpose of the current study was to further evaluate the validity, reliability, and accuracy of this assay for *M*. *leprae* DST in clinical specimens.

**Methodology/Principal findings:**

The specificity and sensitivity for determining the presence and susceptibility of *M*. *leprae* to dapsone based on the *folP1* drug resistance determining region (DRDR), rifampin *(rpoB* DRDR) and ofloxacin (*gyrA* DRDR) was evaluated using 211 clinical specimens from leprosy patients, including 156 multibacillary (MB) and 55 paucibacillary (PB) cases. When comparing the results of qPCR-HRM DST and PCR/direct DNA sequencing, 100% concordance was obtained. The effects of in-house phenol/chloroform extraction versus column-based DNA purification protocols, and that of storage and fixation protocols of specimens for qPCR-HRM DST, were also evaluated. qPCR-HRM results for all DRDR gene assays (*folP1*, *rpoB*, and *gyrA*) were obtained from both MB (154/156; 98.7%) and PB (35/55; 63.3%) patients. All PCR negative specimens were from patients with low numbers of bacilli enumerated by an *M*. *leprae*-specific qPCR. We observed that frozen and formalin-fixed paraffin embedded (FFPE) tissues or archival Fite’s stained slides were suitable for HRM analysis. Among 20 mycobacterial and other skin bacterial species tested, only *M*. *lepromatosis*, highly related to *M*. *leprae*, generated amplicons in the qPCR-HRM DST assay for *folP1* and *rpoB* DRDR targets. Both DNA purification protocols tested were efficient in recovering DNA suitable for HRM analysis. However, 3% of clinical specimens purified using the phenol/chloroform DNA purification protocol gave false drug resistant data. DNA obtained from freshly frozen (n = 172), formalin-fixed paraffin embedded (FFPE) tissues (n = 36) or archival Fite’s stained slides (n = 3) were suitable for qPCR-HRM DST analysis. The HRM-based assay was also able to identify mixed infections of susceptible and resistant *M*. *leprae*. However, to avoid false positives we recommend that clinical specimens be tested for the presence of the *M*. *leprae* using the qPCR-RLEP assay prior to being tested in the qPCR-HRM DST and that all specimens demonstrating drug resistant profiles in this assay be subjected to DNA sequencing.

**Conclusion/Significance:**

Taken together these results further demonstrate the utility of qPCR-HRM DST as an inexpensive screening tool for large-scale drug resistance surveillance in leprosy.

## Author summary

Despite three decades of effective treatment with multidrug therapy (MDT), leprosy persists as a public health problem in many regions of the world. The recent increase in relapse cases, MDT treatment failures, and the emergence of drug-resistant strains of *Mycobacterium leprae* could undermine existing leprosy control measures. PCR/DNA sequencing is currently the method of choice for drug-resistance surveillance in leprosy; however, this technique is not available to most endemic communities and is not cost-effective for large sampling surveys. Therefore, there is a need for drug-susceptibility tests for *M*. *leprae* that could be applicable to large sampling studies, particularly in low-resource areas, where leprosy is endemic, resistance appears to be low, but drug resistance prevalence is likely to be underestimated. Our study demonstrated the utility of qPCR-HRM DST of qPCR-RLEP positive specimens as a reliable screening tool that improves the applicability in endemic regions and reduces cost and time for drug susceptibility screening from a variety of specimen types. To improve the overall reliability we recommend that all mutants be subjected to DNA sequencing, thereby providing valuable information to improve patient treatment outcome and to the global context of leprosy drug resistance monitoring.

## Introduction

Current leprosy control depends solely on case detection and treatment with multi-drug therapy (MDT) including dapsone (DDS), rifampin (RMP) and clofazimine [1]. This strategy is based on the principle that identifying and treating chronic infectious diseases with combinations of bactericidal and bacteriostatic effective antibiotics reduces the bacterial numbers, and limits the emergence and spread of new or existing antibiotic resistant pathogens [2, 3]. Although the rate of relapse following successful completion of the scheduled course of MDT is currently low for both paucibacillary (PB) leprosy (0.1% per year) and multibacillary (MB) leprosy (0.06% per year) [4], several studies have documented drug-resistant *Mycobacterium leprae* strains in relapse cases [5–11] and also the emergence of primary drug resistance [12–15]. However, in contrast to what is known for tuberculosis, the current prevalence of primary and secondary resistance to anti-leprosy drugs is virtually unknown because of the inability to cultivate *M*. *leprae* on axenic medium [16].

Molecular drug susceptibility assays for *M*. *leprae* have been developed for DDS, RMP and ofloxacin (OFX), a second-line drug for treatment of leprosy. These assays are based on PCR amplification and detection of mutant alleles in the drug resistance determining regions (DRDRs) of *folP1*, *rpoB* and *gyrA*, respectively [16]. Among these assays, PCR followed by amplicon DNA sequencing is currently the ‘molecular gold standard’ for drug susceptibility testing (DST) in leprosy [17]. However, this assay format is laborious, expensive and sequencing capabilities are extremely limited in low resource facilities; these are limiting factors for routine drug resistance surveillance as well as prohibitive for large drug resistance sampling surveys, especially in resource-poor countries.

A recent report has described a novel, ‘single-tube’, Real-Time qPCR high resolution melt (HRM) analysis method for anti-leprosy drug susceptibility testing DST [12]. qPCR-HRM DST is based on the amplification of the DRDRs of *folP1*, *rpoB* and *gyrA* genes, and a simple post-PCR step to exploit thermal characteristics of the amplicons for detection of sequence variants. As the amplicons are subjected to high temperatures, the wild type (WT) (drug-susceptible) and mutant (drug-resistant) DRDRs generate distinct HRM profiles. Li et al. (2012) demonstrated a strong correlation between qPCR-HRM DST results and that of PCR/direct DNA sequencing of *M*. *leprae* DRDRs from clinical specimens. It was recommended that DNA be purified using a column-based purification protocols such as DNeasy Blood and Tissue Kit (QIAGEN, Valencia, CA). However, depending on the laboratory, various DNA purification protocols may be implemented to obtain DNA for molecular diagnostic assays. In addition, various types of specimens [e.g. fresh, frozen, formalin-fixed paraffin-embedded (FFPE) or tissues from Fite’s-stained FFPE sections on slides] may serve as the source of *M*. *leprae* DNA.

Therefore, the purpose of the current study was to explore the usefulness of qPCR-HRM DST to predict DDS, RMP and OFX susceptibility in *M*. *leprae* from skin biopsy tissues using either a conventional phenol/chloroform DNA extraction or a column-based DNA purification protocols from fresh-frozen tissues, or FFPE tissue sections, or archival Fite’s-stained FFPE sections on glass slides.

Results of this study support previous reported data that the qPCR-HRM DST assay strongly correlates with PCR/direct DNA sequencing for detection of *M*. *leprae* drug susceptibility directly from clinical specimens. Results also defined the specificity of the assay for *M*. *leprae* as well as demonstrated that conventional phenol based extraction and ethanol precipitation of DNA is a suitable method for this analysis. In addition to ethanol-fixed and fresh specimens, frozen, FFPE sections and Fite’s-stained FFPE sections from glass slides appear to be suitable specimens for this assay.

## Methods

### “Ethics” statement

All procedures involving human subjects including biological sample collections and testing were performed following approval from the governing human subjects’ research ethical committees: in Brazil by the Institutional Review Board at the Federal University of Uberlandia; in the United States by the Institutional Review Board of the Tulane School of Medicine, New Orleans, LA. Written informed consent procedures were carried out in Brazil. In the United States the study was determined to be exempt from consent because archival diagnostic specimens were used, accessed through a de-identified database. Animal procedures were performed under a scientific protocol reviewed and approved by the NHDP Institutional Animal Care and Use Committee (Assurance #A3032-01), and were conducted in accordance with all state and federal laws in adherence with PHS policy and as outlined in *The Guide to Care and Use of Laboratory Animals*, *Eighth Edition*.

### Bacteria and bacterial DNAs

The *M*. *leprae* Thai-53 drug-susceptible reference strain was propagated through serial passage in *nu/nu* mice (Harlan Sprague-Dawley Inc., Indianapolis, IN) and freshly harvested bacilli purified from hind footpads were stored at 4°C and used within 48 hr of harvest, as previously described [18]. Briefly, mice were euthanized by CO_2_ asphyxiation and the hind feet were removed and soaked in 70% ethanol and Betadine to kill surface contaminants. The skin was removed aseptically and the tissue was excised, minced and homogenized in 10 ml of RPMI-10% FBS medium. Tissue debris was removed by slow speed centrifugation (50 x g for 10 sec) and the bacilli-rich supernatant was enumerated by direct counting following staining using the Fite's variation of Ziehl-Neelsen method. The bacteria were resuspended for 8 min in 0.1N NaOH, and washed 3 times in Tris EDTA (TE) buffer to remove extraneous mouse tissue and DNA adsorbed to the bacilli.

A panel of purified *M*. *leprae* DNAs from 19 *M*. *leprae* reference strains (with confirmed drug susceptibility profiles by conventional mouse footpad phenotypic method and genotyping by DNA sequence analysis), including those containing the most common DRDR mutations leading to DDS, RMP and OFX resistance, and susceptible strains, were obtained from the Leprosy Research Centre (LRC), National Institute of Infection Diseases, Tokyo, Japan, the Laboratory of Molecular Biology Applied to Mycobacteria (LABMAM), FIOCRUZ, Rio de Janeiro, and the Institute Lauro de Souza Lima (ILSL), Bauru, Brazil. These DNA samples were used to validate the qPCR-HRM assay for drug susceptibility in *M*. *leprae*.

A panel of DNAs purified (either from clinical specimens isolates or culture) from other mycobacterial strains and bacteria often found in the skin, was obtained from the NHDP-LRB Biobank and used to test the specificity of the qPCR-HRM DST assay. All DNAs were tested for 16S rDNA PCR/direct DNA sequencing to confirm species and as a control for DNA amplification, and for the *M*. *leprae*-specific RLEP quantitative RT-PCR assay [16]. These included: *M*. *lepromatosis (patient specimen)*, *M*. *avium*, *M*. *intracellulare*, *M*. *kansasii*, *M*. *lepraemurium*, *M*. *lufu*, *M*. *marinum*, *M*. *simiae*, *M*. *smegmatis*, BCG-Pasteur, *M*. *ulcerans*, *M*. *bovis*, *M*. *gordonae*, *M*. *fortuitum*, *M*. *haemophilum*, *M*. *tuberculosis*, *Staphylococcus aureus*, *Staphylococcus epidermidis*, *Streptococcus pyogenes*, and *Clostridium perfringens*.

### Human specimens

In this study, 211 skin biopsy specimens obtained from untreated leprosy cases for routine diagnosis of leprosy from Brazil and the U.S. Remaining specimens were “blinded” using a coding system and sent to the NHDP for qPCR-HRM DST for *M*. *leprae*. The code was broken when all data was compiled for final analysis. These specimens were derived from multibacillary (MB; n = 156) and paucibacillary (PB; n = 55) leprosy patients. The procedures for collection and processing of the biopsy specimens are described below.

### DNA purification methods

#### Bacterial DNA

DNA was purified from mouse footpad-derived *M*. *leprae* strain Thai-53 using the DNeasy Blood and Tissue Kit^®^ (QIAGEN, Valencia, CA) and a modified protocol. Briefly, serial dilutions of *M*. *leprae* (starting with 2 x 10^7^) were pelleted at 10,000 x g for 10 min and resuspended in 180 μl Buffer ATL and 20 μl proteinase K, and processed using the DNeasy Blood and Tissue Kit. Samples were incubated for 16 hr at 56°C. Proteinase K was inactivated by incubation at 95°C, 10 min and DNA was purified using the DNA spin column and eluted in 100 μl of AE as per manufacturer’s recommendations.

#### Fresh-frozen skin biopsy DNA

Fresh skin biopsies were obtained from 172 patients and immediately after excision skin biopsies were frozen by immersion into liquid nitrogen and stored at -20°C for up to one week prior to processing (fresh-frozen). Biopsies were finely minced on a sterile petri dish (or glass side) with a single use disposable sterile scalpel or blade and transferred to a vial containing 500 μl of lysis buffer (400 mM NaCl; 50 mM EDTA, pH 8; 25 mM Tris-HCl, pH 8), 30 μl of proteinase K (10 mg/ml) and 40 μl SDS (10%). Tissues were lysed for 12 hr at 56°C and stored until use at -20°C. Proteinase K was inactivated by incubation at 95°C for 10 min and 250 μl Tris-HCl (1M pH 8), 250 μl chloroform/isoamyl alcohol (24:1), and 500 μl Tris buffered phenol (pH 8) were added to each specimen. The solution was homogenized and centrifuged at 10,000 x g for 10 min. The aqueous phase containing DNA was removed and precipitated with an equal volume of ethanol. The pellet was washed with 75% ethanol, followed by a second spin at 10,000 x g for 10 min, air dried, and resuspended in 50 μl of ultrapure water then stored at -20°C.

#### Formalin-fixed paraffin-embedded (FFPE) DNA

DNA was prepared from FFPE skin biopsy tissues from 36 leprosy patients. Briefly, 10 μm thick sections were obtained from paraffin blocks using a microtome. Sections were trimmed of excess paraffin and treated with 1 ml xylene for 30 min. Xylene was removed after centrifugation at 10,000 x g, 10 min, room temperature. Xylene extraction was repeated, xylene was removed and tissues were rinsed in 100%, then 70% ethanol and washed in 1 ml 1x TE Buffer (10 mM Tris-HCL, pH 8.0 and 1 mM EDTA). Tissue was finely minced and added to a 1.5 ml conical microfuge tube containing 180 μl Buffer ATL and 20 μl proteinase K and processed using a modification of the DNeasy Blood and Tissue Kit protocol as described above.

#### Fite’s acid-fast stained slides DNA

DNA was prepared from three archival Fite’s acid-fast stained glass slides containing FFPE sections of skin biopsies from MB leprosy patients. These slides had been stored at room temperature for 1wk, 5 wk and 20 yr, respectively. To remove sealant, slide cover and paraffin from slides, the slides were soaked for 24–48 hr in xylene in separate containers. Coverslips were removed and slides were incubated for an additional 30 min in fresh xylene and rinsed in 100% ethanol and then 70% ethanol. A drop of Buffer ATL was added to the tissue and the tissue was scraped off of the slide with a sterile scalpel and transferred to a 1.5 ml conical microfuge tube containing 180 μl Buffer ATL and 20 μl proteinase K. The DNA was purified using a modification of the DNeasy Blood and Tissue Kit protocol as described above.

### qPCR-RLEP assay

Purified DNA from all types and formats of processed clinical specimens was initially evaluated for the presence of *M*. *leprae* DNA using the quantitative real-time PCR-RLEP assay (qPCR-RLEP) as previously described [19]. Briefly, 2.5 μl aliquots of each specimen were added to PCR master mix and MLRLEP primers and probe (Table 1) in a final volume of 25 μl and tested in the qPCR-RLEP using a standard curve method for quantitation. Standard curve was established using serial 4-fold dilutions of crude cell lysates of mouse footpad-derived *M*. *leprae* after three cycles of freeze-thaw (-80°C, 30 min/98°C, 10 min). Then 1 μl was added to PCR reagents to a 96-well plate and amplified. These dilutions represented 2.0 x 10^7^–4 x 10^3^ *M*. *leprae*/ml equivalents. All samples were run on an ABI 7500 Fast PCR Real-Time System (Applied Biosystems, Foster City, CA) in duplicate.

**Table 1:** Primers and probe used in the comparison of qPCR-HRM DST and PCR/direct DNA sequencing for drug susceptibility testing in *M*. *leprae*.

PCR Assay	Primer/Probe Name	Primer/Probe Sequence 5’-3’	Reference
qPCR-RLEP	MLRLEP-F	GCAGCAGTATCGTGTTAGTGAA	[19]
	MLRLEP Probe	TCGATGATCCGGCCGTCGGCG	
	MLRLEP-R	CGCTAGAAGGTTGCCGTAT	
Mycobacterial 16S rDNA PCR	Myco16S-F	AATTGACGGGGGCCCGCACACAA	[19]
	Myco16S-R	TACGGCTACCTTGTTACGACTTC	
qPCR-HRM DST *folP1*	HRMfolP1-F	GACGTCGGTGGCGAAT	[12]
	HRMfolP1-R	CTCGAGGATCGGTCCTAATGG	
qPCR-HRM DST *rpoB*	HRMrpoB-F	GGTGGTCGCCGCTATCAAGG	[12]
	HRMrpoB-R	CGCTCACGCGACAAACCACC	
qPCR-HRM DST *gyrA*	HRMgyrA-F	CGCTAAGTCAGCACGGTCAGT	[12]
	HRMgyrA-R	CGCACTAACGTGTCATAAATC	
PCR/DNA Sequencing *folP1*	OMSfolP1-F	CTTGATCCTGACGATGCTGT	[17]
	OMSfolP1-R	CCACCAGACACATCGTTGAC	
PCR/DNA Sequencing *rpoB*	OMSrpoB-F	GTCGAGGCGATCACGCCGCA	
	OMSrpoB-R	CAGCAATGAACCGATCAGAC	[17]
PCR/DNA Sequencing *gyrA*	OMSgyrA-F	ATGGTCTCAAACCGGTACATC	[17]
	OMSgyrA-R	TACCCGGCGAACCGAAATTG	

### qPCR-HRM drug susceptibility testing

Drug susceptibility testing of the 19 *M*. *leprae* reference panel of purified DNAs using the q-PCR-HRM DST assay was performed as previously described by Li et al. (2012) with the following modifications. The qPCR reaction included: 10 μl of MeltDoctor HRM Master Mix (Applied Biosystems, Foster City, CA), forward and reverse primers (0.5 μl each of 10 μM stocks) targeting DRDR fragments in the *folP1*, *rpoB* or *gyrA* associated with mutations resulting in DDS, RMP, or OFX resistance, respectively (Table 1), nuclease-free water (8 μl), and (1 μl) of DNA template. Reactions were set up in duplicate in a 96-well PCR plate. Preliminary experiments suggested that it was important to include a negative control (containing no DNA), a drug-susceptible template for target gene being tested, and a mutation control (drug-resistant mutant for gene target being tested) on each plate (each of these in duplicate). The target sequences were amplified using ABI 7500 Fast Real-Time PCR System using the following cycling parameters: 95°C, 2 min; then 45 cycles of 95°C, 10 sec; 60°C, 30 sec; and 72°C, 30 sec. The PCR products were then heated to 95°C, 10 sec and cooled to 60°C over a period of 1 min for hetero-duplex formation. Melting curves for the products were generated by heating the reaction from 60°C to 95°C (at a ramp rate of 0.5°C/sec) according to the instrument default parameters and the fluorescence was automatically recorded at each 0.1°C step.

Post-qPCR HRM analyses of the melt curves were performed using High Resolution Melt Software v3.0.1 (Applied Biosystems, Foster City, CA). The software assembles curves with similar profiles into distinct groups. The curves of the control WT and mutant strains were designated as reference profiles. Data that were similar to the WT reference control were assigned to the drug-susceptible group and data that resembled the mutant reference control were assigned to the “variant” (V) drug-resistant group. For better visualization the melting curves matching each group were color coded.

After standardization, qPCR-HRM DST was performed on DNA from clinical specimens. The software default setting of automatic selection of the melting region (between the pre- and post-melt temperatures), produced acceptable results for reference samples with purified DNA applications. Initially the start and end temperatures of the melting regions were established using these settings. These were then adjusted manually to increase the stringency of the software group assignment, particularly for clinical samples. The baseline settings of the pre-melt and post-melt temperature gates for the qPCR-HRM assay were as follows: *folP1* DRDR pre-melt (80.8°C to 81.3°C) and post-melt (83.4°C to 83.9°C); *rpoB* DRDR pre-melt (85.4°C to 85.9°C) and post-melt (88.1°C to 88.6°C); *gyrA* DRDR pre-melt (81.2°C to 81.6°C) and post-melt (83.4°C to 83.8°C).

### PCR and direct DNA sequencing for drug susceptibility

PCR for *folP1*, *rpoB* and *gyrA* DRDRs was performed on all samples. PCR amplicons corresponding to the DRDRs in *rpoB*, *folP1* and *gyrA* genes were investigated by direct sequencing of PCR amplicons obtained using a modification of the WHO guidelines for Global Surveillance of Drug Resistance in Leprosy [17]. Since OFX resistance in leprosy is of a lesser concern, the *gyrA* DRDR sequence was obtained from a subset (80%) of the samples. Briefly, the PCR reaction mix included 25 μl of AmpliTaq Gold 360 PCR Master Mix (Applied Biosystems, Foster City, CA), forward and reverse primers (2.5 μl each of 10 μM stocks), nuclease-free water (15 μl), and DNA from samples (5 μl). PCR cycling parameters were: 95°C for 2 min followed by 45 cycles of 95°C for 10 sec, 58°C for 30 sec, and 72°C for 30 sec and then a final extension step at 72°C for 7 min. PCR products were loaded in 2% agarose gel for confirmation of the fragment amplification. Amplified products were purified through QIAquick PCR Purification Kit (QIAGEN). DNA concentrations were determined using NanoDrop 8000 spectrophotometer (Thermo Fisher Scientific Inc., Wilmington, DE) and sequenced by capillary electrophoresis using BigDye Terminator v3.1 cycle sequencing kit in the ABI Prism 3130 Genetic Analyzer (Applied Biosystems). Sequence data was analyzed using the nucleotide database in the Basic Local Alignment Search Tool (BLAST) (http://blast.ncbi.nlm.nih.gov) to identify mutations associated with drug resistance.

## Results

### Performance evaluation and standardization with reference strains

**Fig 1:**
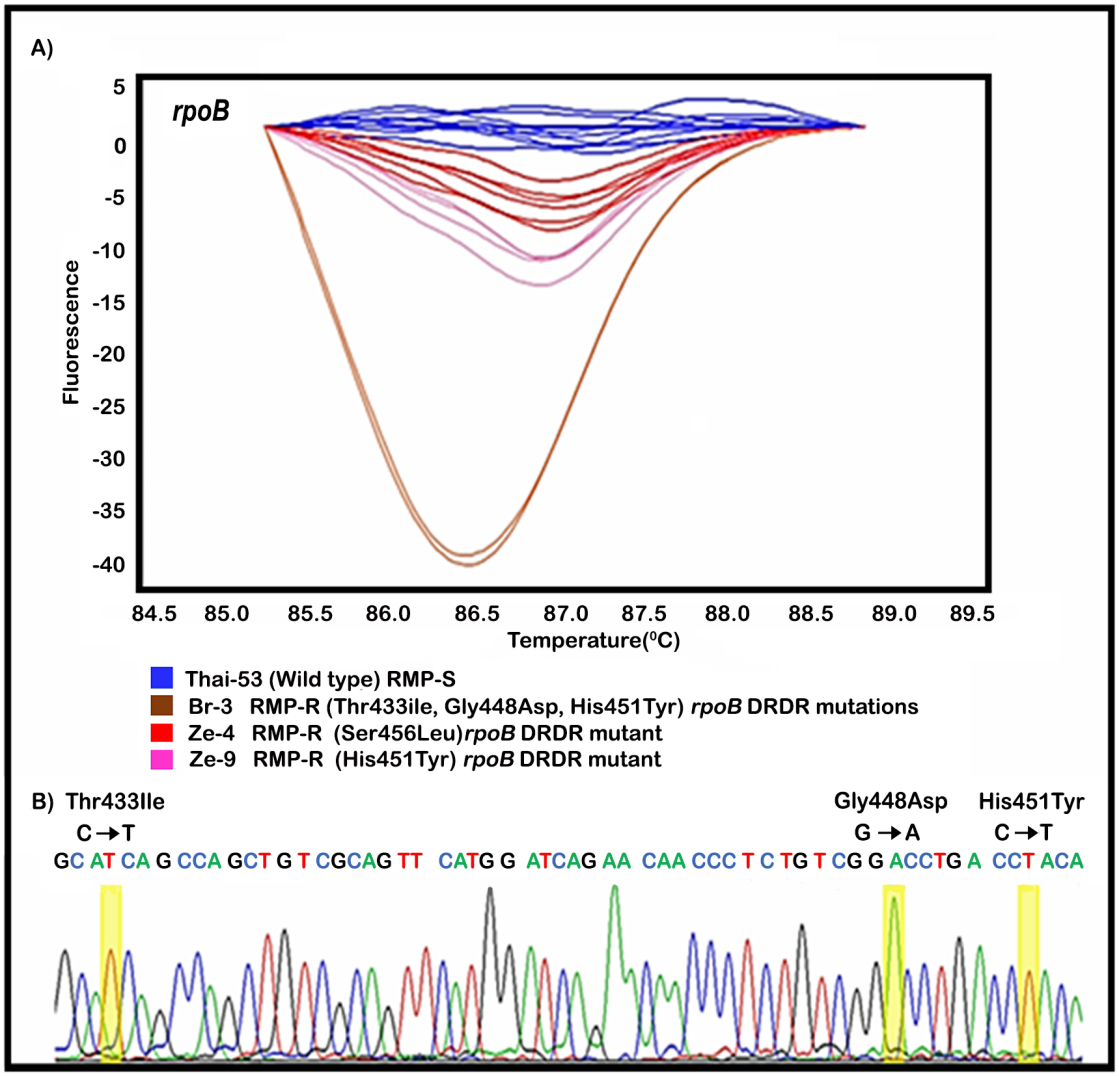
Comparison of *M*. *leprae* qPCR-HRM DST and PCR/DNA sequencing DST results of RMP-resistant *M*. *leprae* Br-3. A) Difference plots graphic display of the post-qPCR HRM analysis of the *rpoB* drug resistance determining region (DRDR) of *M*. *leprae* Br-3. The DNA melting curves obtained in the analysis of mutant strains, deviate from the wild type profile (Thai-53 strain, shown in blue color). B) DNA chromatogram result for the DNA sequence of *rpoB* DRDR of *M*. *leprae* Br-3 sample, showing three independent mutations associated with RMP resistance.

qPCR-HRM DST results from the analysis of 19 drug-susceptible (n = 5) and drug-resistant (n = 14) reference strains demonstrated that all characterized drug-resistant strains had distinct variant (“V”) HRM assignment group based on melt curve profiles from that of the drug-susceptible, WT *M*. *leprae* Thai-53 type strain (Table 2). In addition, all drug-susceptible reference strains generated the WT HRM profile. The genotypes of these strains were confirmed by DNA sequencing of the DRDRs. Interestingly, one strain (Br-3) showed multiple missense mutations within the *rpoB* DRDR (Fig 1). The specificity of qPCR-HRM for detection of mutations in the *M*. *leprae* DRDRs for DDS, RMP and OFX was 100%.

**Table 2:** qPCR/High Resolution Melt (HRM) drug susceptibility testing (DST) of *M*. *leprae* reference strains.

	Drug Resistance Determining Region					
Reference strain	*folP1*		*rpoB*		*gyrA*	
	HRM profile^a^	PCR/DNA sequencing^b^	HRM profile	PCR/DNA sequencing	HRM profile	PCR/DNA sequencing
**Drug-resistant**						
Ai-3	V	T(ACC)53I(ATC)^c^	WT	WT	WT	WT
Am-1	V	P(CCC)55L(CTC)	WT	WT	WT	WT
Ho-4	V	P(CCC)55L(CTC)	V	S(TCG)456L(TTG)	V	A(GCA)91V(GTA)
Ku-3	V	T(ACC)53I(ATC)	WT	WT	WT	WT
Ku-6	V	P(CCC)55L(CTC)	V	D(GAT)441Y(TAT)	WT	WT
Ry-6	WT	WT	WT	WT	V	A(GCA)91V(GTA)
Ze-2	V	P(CCC)55L(CTC)	WT	WT	WT	WT
Ze-4	V	T(ACC)53I(ATC)	V	S(TCG)456L(TTG)	V	A(GCA)91V(GTA) & WT
Ze-5	V	P(CCC)55L(CTC);	V	S(TCG)456L(TTG)	WT	WT
		WT53 & T(ACC)53I(CTC)				
Ze-9	V	P(CCC)55L(CTC)	V	H(CAC)451Y(TAC)	WT	WT
Br-2	V	P(CCC)55R(CGC)	V	S(TCG)456M(ATG)	WT	WT
Br-4	V	P(CCC)55R(CGC)	V	S(TCG)456L(TTG)	WT	WT
Br-5	V	P(CCC)55R(CGC)	V	S(TCG)456L(TTG)	WT	WT
Br-3	V	P(CCC)55L(CTC)	V	T(ACC)433I(ATC); G(GGC)448D(GAC) H(CAC)451Y(TAC)	V	A(GCA)91V(GTA)
**Drug- Susceptible**						
Ai-2	WT	WT	WT	WT	WT	WT
Iz-1	WT	WT	WT	WT	WT	WT
Ke-4	WT	WT	WT	WT	WT	WT
Ky-2	WT	WT	WT	WT	WT	WT
Thai-53	WT	WT	WT	WT	WT	WT

^a^ qPCR-HRM DST profiles for drug resistance determining regions (DRDR) of *M*. *leprae; folP1* for dapsone, *rpoB* for rifampin and *gyrA* for ofloxacin susceptibility. WT = wild type, consistent with the drug-susceptible phenotype; V = HRM variant, consistent with the drug-resistant phenotype of *M*. *leprae*

^b^DNA sequence for DRDR of *M*. *leprae- folP1* for dapsone, *rpoB* for rifampin, and *gyrA* for ofloxacin.

^c^Drug resistant mutation (e.g. T(ACC)53I(ATC) = A missence mutation has occurred in codon 53 of the *folP1* gene resulting in the substitution of a isoleucine (I) amino acid residue for a threonine (T) residue in the encoded dihydopteroate synthase protein of this *M*. *leprae* strain.

**Fig 2:**
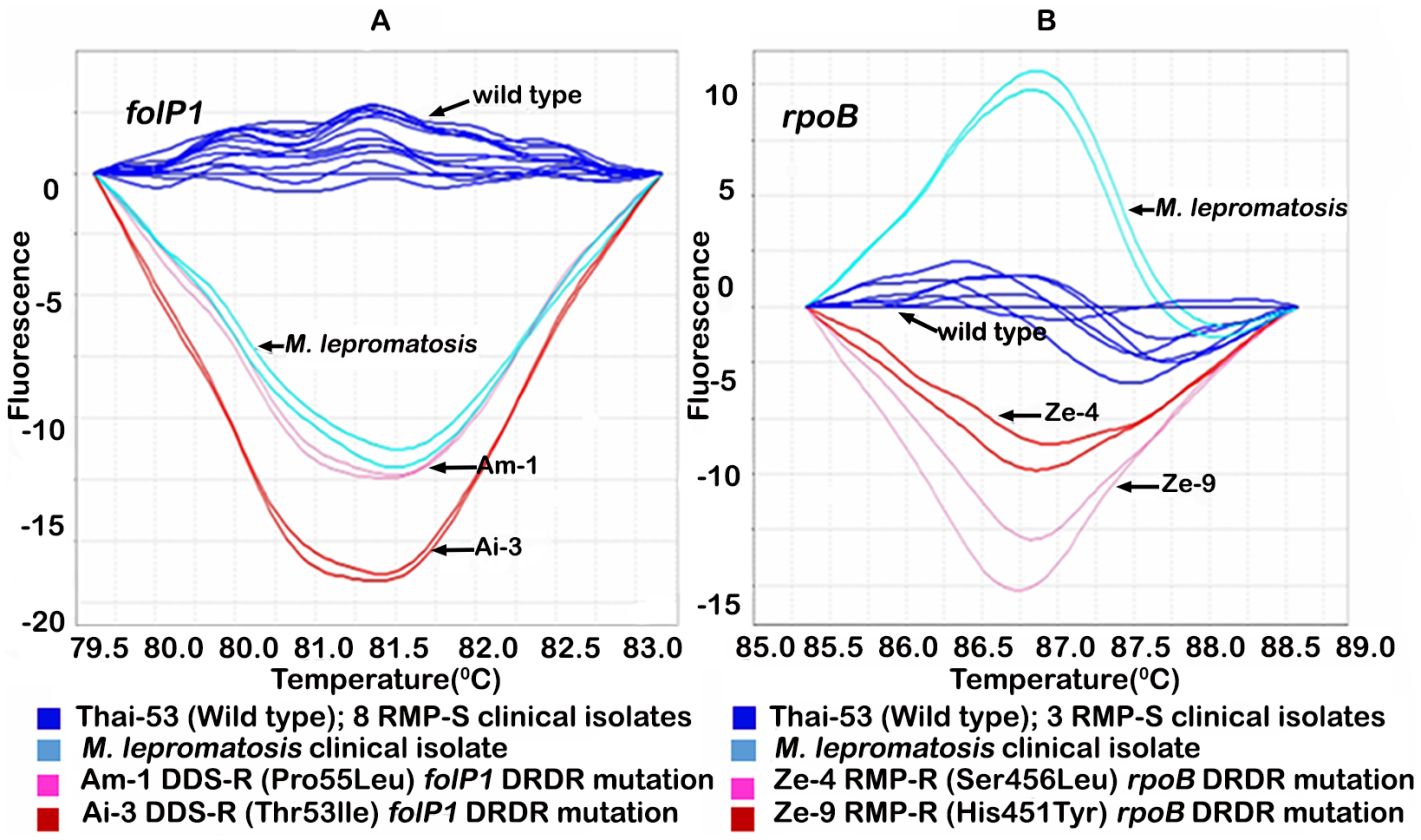
Comparison of *M*. *leprae* qPCR-HRM DST difference plots graphic displays of the post-qPCR HRM analyses of the *rpoB* and *folP1* drug resistance determining regions (DRDR) of *M*. *lepromatosis* and *M*. *leprae* for DDS and RMP susceptibility. DNA melting curves, obtained from the analysis of strains, deviate from the wild-type profile (Thai-53 strain, shown in dark blue color); A) *folP1* DRDR HRM profiles and B) *rpoB* DRDR HRM profiles.

The specificity of the qPCR-HRM DST for detection of *M*. *leprae* was defined using purified DNA from a variety of other mycobacterial and bacterial species. Results demonstrated that only *M*. *lepromatosis* generated a PCR amplicon in the qPCR-HRM in *folP1* and *rpoB* DRDRs, with no amplification in *gyrA*. In each of these two assays *M*. *lepromatosis* DRDR was assigned to a distinct “V” HRM profile from that of *M*. *leprae* (Fig 2). Alignment of DRDRs of *folP1* and *rpoB* from *M*. *leprae* and *M*. *lepromatosis* demonstrated multiple base-pair mismatches between these two strains (data not shown). It is of note that RLEP results demonstrated that *M*. *lepromatosis* presented no DNA amplification for the *M*. *leprae*-specific gene fragment, as observed for all mycobacterial and bacterial species tested by qPCR-RLEP.

### Drug susceptibility testing of clinical samples

The *M*. *leprae* qPCR-HRM DST for DDS, RMP and OFX was conducted on 211 RLEP positive clinical specimens preserved using a variety of procedures including: freezing, FFPE, and Fite’s-stained FFPE sections on glass slides and processed using either conventional or column-based DNA purification protocols. Results demonstrated that 6/14 (42.9%) TT and 29/41 (70.7%) BT patient specimens and 154/156 (98.7%) MB specimens gave HRM results for all three drugs (S1 Table). The two negative specimens from the MB group were classified as BB patients (B-154, B-156; S1 Table) and contained low numbers of bacteria as defined by qPCR RLEP. In contrast, amplicons for all three DRDRs were obtained from 4/14 (28.6%) TT, 26/41 (63.4%) BT and 143/156 (91.7%) MB patient specimens using standard PCR assays for DNA sequencing. Although some specimens with as little as 33 *M*. *leprae*/ml (enumerated by qPCR RLEP assay) did yield HRM results, the majority of those containing ≤ 350 *M*. *leprae*/ml did not yield reliable results in the all three qPCR-HRM DST assays.

When HRM profiles were initially compared to DNA sequencing results there was a 97% correlation between the two assays. Five specimens giving a “V” HRM profile in the qPCR-HRM assay gave the susceptible genotype in DNA sequencing (B-07, B-17, B-47, B-125, and B-172; S1 Table). These samples were originally extracted using a non-column-based DNA purification protocol. These samples were subsequently re-extracted using the DNeasy Blood and Tissue kit. DNA was then subjected to qPCR-HRM DST. Wild-type HRM profiles were observed for all of these specimens.

**Fig 3:**
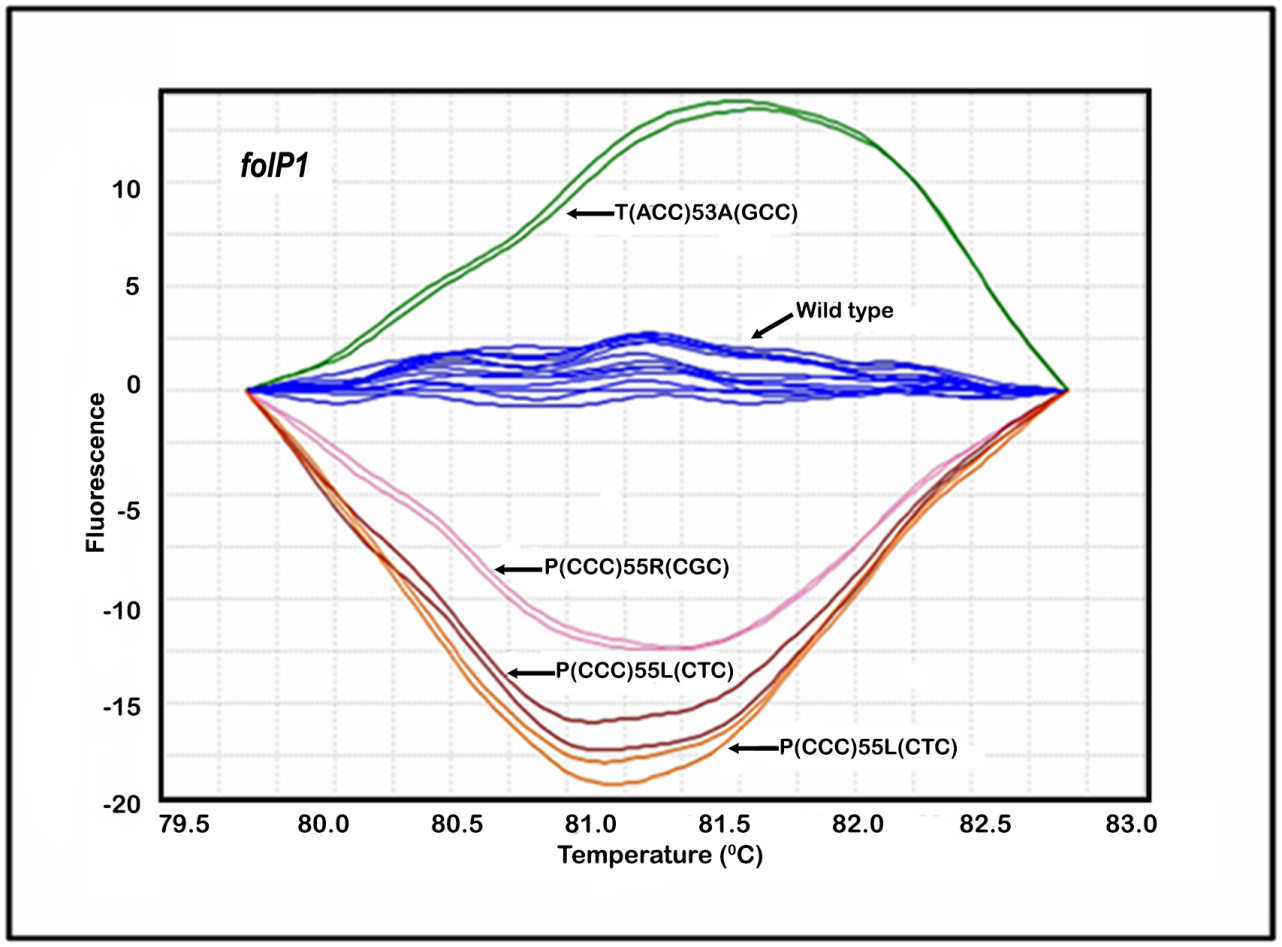
Comparison of *M*. *leprae* qPCR-HRM DST difference plot graphic displays of the post-qPCR HRM analyses of the *folP1* from clinical strains. DDS-resistant strains: T(ACC)53A(GCC) = Ref ID# N-33; P(CCC)55R(CGC) = Ref ID# N-08; T(ACC)53I(ATC) = Ref ID# N-30; P(CCC)55L(CTC) = Ref ID# N-21. *M*. *leprae* DDS-susceptible strains: Wild-type = Thai-53 (control); Ref ID# B-01; Ref ID# B-08; Ref ID# B-11; Ref ID# B-23; Ref ID# B-32; and Ref ID# B-55.

Four additional clinical specimens (N-08, N-21, N-30 and N-33) generated distinct “V” HRM profiles in *folP1* DRDR (Table 3). These mutations have been previously associated with DDS-R leprosy. One of these mutations (ACC→GCC) resulted in the substitution of an alanine amino acid residue for a threonine in the dihydropteroate synthase of *M*. *leprae* encoded by *folP1*. This particular mutation increased the melting temperature (Tm) 0.2°C, which generated a HRM difference in fluorescence plot as a melting curve shape above the WT profile (Fig 3). This is a characteristic that has not been observed in any other SNP mutation in the *folP1* DRDR associated with the DDS-R genotype evaluated thus far. One of the four DDS-R specimens also generated a “V” HRM profile for the *rpoB* DRDR, consistent with RMP-R *M*. *leprae* phenotype (Table 3). All specimens tested in the qPCR-HRM *gyrA* assay generated the WT for the *gyrA* DRDR, consistent with OFX-susceptible *M*. *leprae* genotype (S1 Table).

**Table 3:** *M*. *leprae* drug-resistant mutants in clinical specimens from leprosy patients identified using molecular drug susceptibility assays.

*M*. *leprae* Ref #	Drug Resistance Determining Region					
	*folP1*		*rpoB*		*gyrA*	
	HRM Profile^a^	PCR/DNA Sequencing^b^	HRM Profile	PCR/DNA Sequencing	HRM Profile	PCR/DNA Sequencing
N-08	V	P(CCC)55R(CGC) ^c^	WT	WT	WT	WT
N-21	V	P(CCC)55L(CTC)	WT	WT	WT	WT
N-33	V	T(ACC)53A(GCC)	V	S(TCG)456L(TTG)	WT	WT
N-30	V	T(ACC)53I(ATC)	WT	WT	WT	WT

^a^HRM profile = profile generated from qPCR-HRM DST: V = variant containing mutation (drug-resistant phenotype); WT = wild-type sequence (drug-susceptible phenotype)

^b^PCR/DNA Sequencing = DNA sequence of drug resistance determining region for each target gene.

^c^Drug resistant mutation (e.g., T(ACC)53I(ATC) = A missense mutation has occurred in codon 53 of the *folP1* gene resulting in the substitution of a isoleucine (I) amino acid residue for a threonine (T) residue in the encoded dihydopteroate synthase protein of this *M*. *leprae* strain.

### Detection of mixed *M*. *leprae* infections

**Fig 4:**
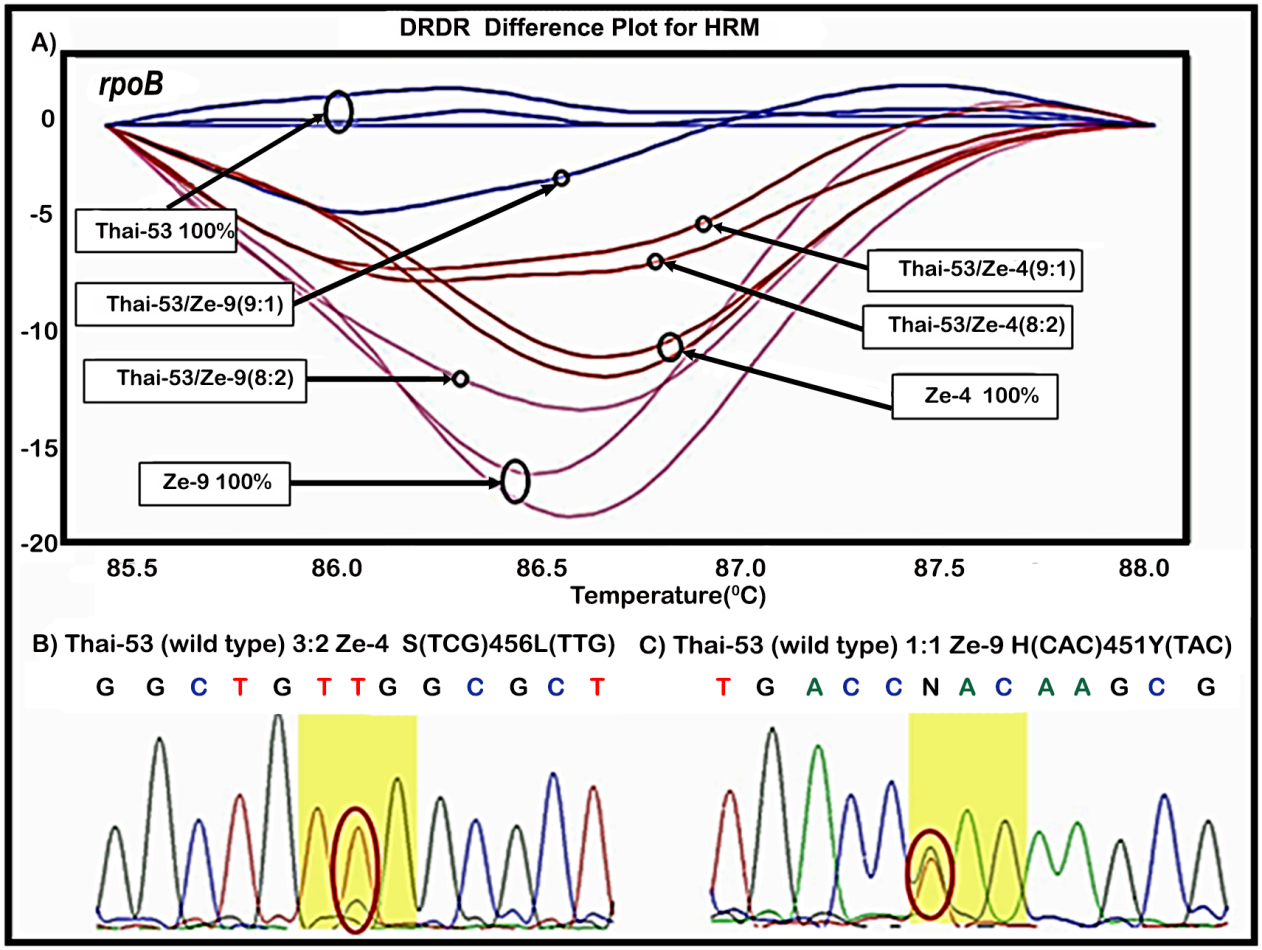
Determination of RMP-resistant mutant allele detection limit in a mixture of wild-type/RMP-resistant mutant DRDRs using *M*. *leprae* qPCR-HRM DST. A) Difference plot graphic displays of the post-PCR HRM melting curve analysis of the *rpoB* drug resistance determining region (DRDR) of wild-type (Thai-53) and RMP-R strains (Ze-4 and Ze-9) and mixtures of Thai-53 and these RMP-R mutants; B) PCR-DNA sequencing of the *rpoB* DRDRs of 3:2 ratio of Ze-4; and C) PCR-direct DNA sequencing of the *rpoB* DRDRs of 1:1 ratio of Ze-9. Yellow highlights identify codon where mutations were observed.

To further test the ability of qPCR-HRM DST to detect a mixed infection of drug-resistant mutants in a background of drug-susceptible *M*. *leprae*, RMP-R and RMP-S DNAs were combined using 10-fold dilutions of templates resulting in ratios 9:1 (WT:RMP-R) to 9:1 (RMP-R:WT) ratios. The DNA mixtures containing equimolar DNA concentrations were analyzed by both qPCR-HRM DST and PCR/direct DNA sequencing. Results demonstrated that the qPCR-HRM DST for RMP could detect as little as 10% of the RMP-R genotype in a background of 90% RMP-S genotype (Fig 4A). In comparison, the minimal concentration of the RMP-R genotype to effectively detect a mixed allele by PCR/direct DNA sequencing was 30% (Fig 4B and 4C).

## Discussion

The control of leprosy relies solely on early case detection and treatment. The success of MDT is also critical for preventing morbidities and disabilities associated with infection. Even though there are relatively low levels of relapse reported after MDT, the nearly stable incidence rate attests to continuing disease transmission. Studies have also documented the emergence of drug-resistant *M*. *leprae* strains in relapse cases [5–11, 14]. However, the current prevalence of both primary and secondary resistance to anti-leprosy drugs is virtually unknown because of the inability to cultivate *M*. *leprae* on axenic medium and the limited availability of molecular DST in laboratories in low resource, highly endemic countries. The use of real-time PCR in combination with high-resolution melt technologies has been recently reported for DST of *M*. *leprae* to DDS, RMP and OFX [12], which can increase throughput, reduce costs, and support the inclusion of more patients, including new and relapse cases. It is now possible to discriminate drug-resistant mutant loci by post-PCR analysis of the variations in the double-strand DNA dissociation temperatures of amplicon melting curves. The suggested requirements for qPCR-HRM are a highly purified DNA template, a compatible Real-Time PCR thermalcycler, a PCR mix containing appropriate enzymes, buffer, DNA-saturating dyes, and high-resolution melt software. For mutation analysis by HRM, there are no operator-dependent sample manipulations after the qPCR is assembled and no need for additional reagents. In addition, the HRM does not require allele-specific primers or expensive probes for detection of mutations associated with resistance of *M*. *leprae* to DDS, RMP and OFX in clinical specimens. It has been also strongly recommended that column-based DNA purification protocols be used to provide suitable template for HRM-based DST when frozen, ethanol-fixed clinical specimens were evaluated. In the current study we extended these observations by evaluating the effect of other DNA purification and specimen fixation protocols on HRM analysis. In addition, the specificity of qPCR-HRM DST of *M*. *leprae* for DDS, RMP and OFX was defined using other mycobacterial and bacterial DNAs and clinical specimens having mycobacterial infections.

To standardize the qPCR-HRM DST of *M*. *leprae* for DDS, RMP and OFX for this study, a library of drug-resistant *M*. *leprae* isolates containing several mutations in each of three drug target genes (*folP1*, *rpoB* and *gyrA*, respectively) was received from the LRC laboratory and used to establish the qPCR-HRM DST assay using the ABI 7500 Fast Real-Time PCR Instrument and ABI HRM software. In addition, four additional multi-drug-resistant isolates from the LABMAM laboratory were added to this library. Even though these mutants had not been previously evaluated in qPCR-HRM DST, our results demonstrated that all mutant strains within this combined collection were correctly identified by this technique. The mutations associated with drug resistance in the *rpoB*, *folP1* and *gyrA* DRDRs of *M*. *leprae* have been associated with moderate to high levels of drug resistance in *M*. *leprae* using mouse footpad DST. No mutations have been identified in low level dapsone-resistant *M*. *leprae*.

For DDS resistance *folP1* missense mutations within codons 53 and 55 have been associated with the development of resistance [20, 21]. We identified *folP1* mutation types (ACC→ATC) in codon 53 and (CCC→CTC and CCC→CGC) in codon 55. In addition, one of four clinical specimens generating a “V” *folP1* profile in the current study contained a *folP1* mutation type (ACC→GCC) in codon 53. Its HRM curve was located above and right of that of the WT strains. This was most likely due to the substitution of a guanine base for an adenine base in codon 53 of the *folP1* of this *M*. *leprae* strain, resulting in an increased melting temperature of its DNA heteroduplex. Together, these four *folP1* mutation types cover 91% of the DDS-R mutants described worldwide [16, 22]. The HRM profile of *folP1* mutation type (CCC→CGC) in codon 55 was found in three characterized Brazilian DDS-R isolates. This mutant was difficult to discern by HRM, as it was initially assigned to the WT group. This was most likely due to the minimal change in the melting temperature of its duplex containing a substitution of a guanine base for a cytosine base. It was necessary to adjust software parameters for the gate region between the pre- and post-melt temperatures in order for this mutant to be grouped as a “V” profile. Thereafter, these adjustments defined the baseline settings as described in the methods section. The presence of double *folP1* mutation types (ACC→CTC) in codon 53 and (CCC→CTC) in codon 55, as well as, a mixed infection with WT type allele in codon 53 was also confirmed in one isolate. Together these data confirmed earlier observations that the q-PCR-HRM DST was highly specific for detection of DDS susceptibility in *M*. *leprae* [12].

For RMP resistance, *rpoB* mutation types (ACC→ATC) in codon 433, (GGC→CAC) in codon 448, (CAC→TAC) in codon 451, and (TCG→TTG) in codon 456 have been previously associated with the RMP-R phenotype of *M*. *leprae* and have previously shown to generate distinct “V” HRM profiles [12]. Results from the current study support these observations and represent over 80% of the RMP-R mutants described worldwide[16]. In addition, one Brazilian RMP-R isolate that generated a distinct “V” HRM *rpoB* profile contained multiple *rpoB* mutation types including: (ACC→ATC) in codon 433, (GGC→CAC) in codon 448, and (CAC→TAC) in codon 451. In addition, this isolate also had resistance to DDS (*folP1* mutation type CCC→CTC in codon 55) and OFX (*gyrA* mutation type GCA→GTA in codon 91). qPCR-HRM DST *gyrA* confirmed initial results that qPCR-HRM DST detects a mutation in codon 91 (GCA→GTA) in clinical isolates; which occurs in more than 90% of the reported mutations associated with the development of OFX resistance in *M*. *leprae* [16, 23]. The HRM analysis was also sensitive in detecting the low levels of mutant alleles in the *folP1*, *rpoB* and *gyrA* DRDRs in mixed infections of WT and resistant *M*. *leprae*. For example, in strain Ze-4 the melting curve for *gyrA* DRDR differed from that of the WT and the expected mutant. This was assigned to a “V” profile. DNA sequencing analysis of this mutant confirmed mixed alleles in codon 91 of the *gyrA*. In addition, the mixed infection of DDS-R and DDS-S *M*. *leprae* was also confirmed in Ze-5. Thus, these results reinforce that HRM clustering can be sensitive to the presence of multiple alleles, as reported by Li et al. (2012).

Further analysis of mixed alleles demonstrated that as little as 10% resistant *M*. *leprae* genotype in 90% susceptible genotype was sufficient to convert the WT profile to “V” profile in the HRM assay for DST. In contrast, DNA sequencing DST required as much as 30% of the resistant allele for detection of the mixed genotypes. This confirms that qPCR-HRM DST analysis may enable detection of minor populations of mutant alleles in a WT background and thus the emergence of drug resistance.

Taken together, these results confirmed the initial observations of Li et al. (2012), demonstrating a strong correlation between qPCR-HRM DST results and that of PCR/DNA sequencing from characterized isolates. Future studies to explore the HRM capability in genotyping lower proportions of drug-resistant strains in mixed infections within the same patient are needed to understand the clonal complexity in the course of *M*. *leprae* infection, possibly in animal models experiments. This qPCR-HRM strategy for DST directly from clinical samples, could be potentially optimized to analyze RMP-R in *M*. *tuberculosis*, once it has been demonstrated that the conventional *in vitro* culture methods may allow one strain to predominate, hindering the detection of resistant strains [24].

qPCR-HRM DST results were obtained for all three drugs from 99% of all MB leprosy patient biopsies in which *M*. *leprae* DNA was detected using qPCR RLEP. The two MB specimens that did not produce qPCR-HRM DST results were from mid-borderline (BB) leprosy patients with low bacterial numbers. Four of the clinical specimens from MB patients generated “V” HRM profiles consistent with DDS-R leprosy. One of these specimens also contained an *rpoB* “V” HRM profile. qPCR-HRM DST results were obtained for all three drugs from 63% of all PB leprosy patient biopsies in which *M*. *leprae* DNA was detected using qPCR RLEP. Although results were obtained from PB specimens with as little as 30 *M*. *leprae* (enumerated by qPCR-RLEP), more consistent results were obtained from samples with ≥ 350 bacteria. No drug resistance was detected in the PB group. DNA sequencing confirmed the genotypes of these clinical specimens and our data confirm the initial report of Li et al. [12].

Several other key factors were critical to the success of qPCR-HRM DST of *M*. *leprae* directly from clinical specimens. The first of these was the purity of the DNA in the samples to be analyzed for HRM analysis. A column-based DNA purification protocol has been recommended for sample processing for qPCR-HRM DST analysis [12]. However, our results demonstrated that the vast majority (97%) of DNA samples prepared from skin biopsy tissues using a conventional DNA purification protocol (Proteinase K treatment, phenol/chloroform extraction and ethanol precipitation) provided suitable template for qPCR-HRM DST. While we concur that column-based DNA purification provides the best quality template for HRM, it is important to note that the conventional DNA purification protocol is also suitable for HRM as a low cost alternative for resource poor laboratories. However, we also recommend that qPCR-RLEP be performed on all specimens prior to qPCR-HRM DST for leprosy as well as all specimens with "V" HRM profiles be sequenced.

The initial study also evaluated ethanol-fixed skin biopsy tissues as a source of DNA for the qPCR-HRM DST [12]. The current study extended this analysis to examine the effect of other methods for specimen storage or fixation on the qPCR-HRM DST. These included fresh-frozen specimens, specimens recovered from FFPE tissue sections, and from archival Fite’s stained formalin-fixed paraffin-embedded (FFPE) sections from glass slides. Fite’s stain is a modification of the Ziehl-Neelsen acid-fast staining procedure that preserves the precarious acid fastness of *M*. *leprae* and thereby is used extensively to stain for detection of *M*. *leprae*. Results demonstrated that the method of fixation does not appear to have an effect on the ability to generate qPCR-HRM DST results. The number of bacilli (~ 350) appears to be the limiting factor. Therefore, combining these data with that of previous published data suggest that tissues preserved for histopathology (FFPE blocks, slides for histopathology), ethanol-fixed and fresh-frozen tissues are all suitable specimens for qPCR-HRM DST of *M*. *leprae*.

Another key factor for qPCR-HRM DST of *M*. *leprae* directly in clinical specimens is the specificity of the qPCR amplification for *M*. *leprae* DRDRs. Since the dye used in this assay binds nonspecifically to dsDNA and therefore, any dsDNA PCR product can emit fluorescence, the current study characterized the specificity of the qPCR-HRM DST assays for *M*. *leprae*. After testing 20 other bacterial and mycobacterial DNAs and biopsy specimens containing some of these mycobacterial species (*M*. *avium*, *M*. *haemophilum*, *M*. *gordonae*, *and M*. *lepromatosis*), *M*. *lepromatosis* was the only other mycobacterial species that generated amplicons in qPCR HRM DST. This was not surprising because *M*. *leprae* and *M*. *lepromatosis* are highly related mycobacterial species which both cause leprosy and are now referred to as the Leprosy Complex [25]. Analysis of the primer sequences for the RT-PCR HRM assays confirmed a high degree of homology of these primers to that of *M*. *lepromatosis* DRDRs (S1 Fig). HRM analysis of these amplicons generated “V” HRM profiles distinct from but similar to that found for some drug-resistant *M*. *leprae* strains. However, this appeared to be due to mutations in other codons within the DRDRs of *M*. *lepromatosis* not associated with resistance in *M*. *leprae*. To avoid this potential specificity problem with *M*. *lepromatosis*, we recommend that all clinical specimens be “positive” in the qPCR-RLEP assay prior to being analyzed by qPCR-HRM DST. In addition, all specimens with a "V" HRM profile should be subjected to DNA sequencing.

Proper gating of the HRM data for the difference plots was another key factor affecting the performance of the qPCR-HRM DST using clinical specimens. This was vastly improved by the addition of two dilutions of an appropriate known drug-susceptible and -resistant control DNAs on each qPCR-HRM plate. These controls consisted of the DRDRs of: Ze-4 containing the high frequency rpoB mutant allele S(CTG)456L(TTG); Ai-3 containing the high frequency *folP1* mutant allele, T(ACC)53I(ATC); and Ry-6 containing the high frequency *gyrA* mutant allele, A(GCA)91V(GTA). Control samples were critical to establish reproducible derivative melting curve plots, which could then be used as reference peaks at the expected melting temperature for each specific DRDR fragment of *M*. *leprae*. The appropriate gate for each allele could be set and therefore further define the parameters for assigning WT and “V” profiles of the unknowns. In addition, to prevent the interference with the analysis of other samples on the plate, any sample that did not amplify before the Ct value = 36 in the respective qPCR assay for each of the DRDRs or that did not give the expected peak in the derivative melt curve plot were excluded from analysis.

In summary the qPCR-HRM DST is a ‘single-tube’ assay that can identify genetic variants in drug “target” genes by post-PCR analysis of the shapes and melting temperatures of amplicon melting curves. Since PCR amplicons and melting curves are generated in the same instrument, there are no operator-dependent sample manipulations after the qPCR reaction is assembled and no need for additional reagents or supplies to determine drug susceptibility. This reduces the risk of amplicon cross contamination and the cost of DNA sequencing. In addition, 42 samples (tested in duplicate) can be investigated at the same time in a single 96-well plate within 3 hr. Therefore, qPCR-HRM DST lends itself to high throughput screening of leprosy drug resistance. The estimated cost of a single reaction using qPCR-HRM DST is ~ $3 (U.S. dollars). In contrast, PCR/DNA sequencing DST includes post PCR amplicon purification and quantification prior to DNA sequencing. The estimated cost for analysis of a single specimen is $22 (U.S. dollars). These data strongly suggest that qPCR-HRM DST for *M*. *leprae* has broad applicability and can dramatically reduce the cost and time involved with DNA sequencing by only sequencing those specimens that generate the “V” HRM profile, especially in resource poor endemic regions thereby providing valuable information to improve patient treatment outcome and to aid in the global context of leprosy drug resistance.

## Supporting information

S1 Table Molecular drug susceptibility testing of *M*. *leprae* from clinical specimens. (XLSX)

S1 Fig Alignment of the drug resistance determining regions of DNA-dependent RNA polymerase β-subunit (*rpoB*) genes *from Mycobacterium leprae* (Thai-53) and *Mycobacterium lepromatosis* (FJ924). Yellow colored bases denote *rpoB* DRDR primer sequences in *M*. *leprae*. Green colored bases denote *rpoB* codons that are associated with rifampin resistance in *M*. *leprae*.
(TIF)
