# Supplementary figures and images for: qPCR-High resolution melt analysis for drug susceptibility testing of Mycobacterium leprae directly from clinical specimens of leprosy patients
Source: PLoS Negl Trop Dis. 2017 Jun 1;11(6):e0005506. doi: 10.1371/journal.pntd.0005506 (PMC5453413; doi:10.1371/journal.pntd.0005506)

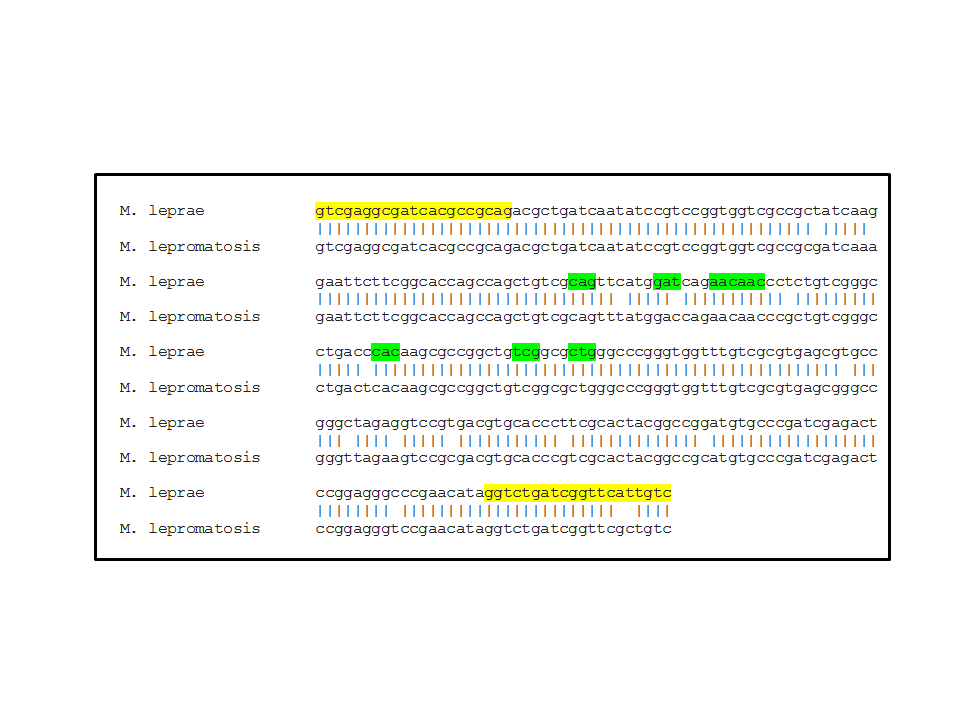

Supplement: S1 Fig — Yellow colored bases denote rpoB DRDR primer sequences in M. leprae. Green colored bases denote rpoB codons that are associated with rifampin resistance in M. leprae. (TIF) [file pntd.0005506.s002.tif]
